# A molecular genetic toolbox for *Yarrowia lipolytica*

**DOI:** 10.1186/s13068-016-0687-7

**Published:** 2017-01-03

**Authors:** Erin L. Bredeweg, Kyle R. Pomraning, Ziyu Dai, Jens Nielsen, Eduard J. Kerkhoven, Scott E. Baker

**Affiliations:** 1 Earth and Biological Sciences Directorate, Environmental Molecular Sciences Laboratory, Richland, WA 99354 USA; 2 Chemical & Biological Process Development Group, Energy and Environment Directorate, Pacific Northwest National Laboratories, Richland, WA 99354 USA; 3 Systems and Synthetic Biology, Department of Biology and Biological Engineering, Chalmers University of Technology, Göteborg, Sweden; 4 Novo Nordisk Foundation Center for Biosustainability, Technical University of Denmark, Hørsholm, Denmark; 5 Department of Energy, Battelle EMSL, 3335 Innovation Blvd, Richland, WA 99354 USA

**Keywords:** *Yarrowia lipolytica*, GFP localization, Overexpression plasmid, Genome sequence, Tools, Superfolder GFP, Hygromycin B, Protein tagging, Organelle labeling, Isogenic

## Abstract

**Background:**

*Yarrowia lipolytica* is an ascomycete yeast used in biotechnological research for its abilities to secrete high concentrations of proteins and accumulate lipids. Genetic tools have been made in a variety of backgrounds with varying similarity to a comprehensively sequenced strain.

**Results:**

We have developed a set of genetic and molecular tools in order to expand capabilities of *Y. lipolytica* for both biological research and industrial bioengineering applications. In this work, we generated a set of isogenic auxotrophic strains with decreased non-homologous end joining for targeted DNA incorporation. Genome sequencing, assembly, and annotation of this genetic background uncovers previously unidentified genes in *Y. lipolytica*. To complement these strains, we constructed plasmids with *Y. lipolytica*-optimized superfolder GFP for targeted overexpression and fluorescent tagging. We used these tools to build the “*Yarrowia lipolytica* Cell Atlas,” a collection of strains with endogenous fluorescently tagged organelles in the same genetic background, in order to define organelle morphology in live cells.

**Conclusions:**

These molecular and isogenetic tools are useful for live assessment of organelle-specific protein expression, and for localization of lipid biosynthetic enzymes or other proteins in *Y. lipolytica*. This work provides the Yarrowia community with tools for cell biology and metabolism research in *Y. lipolytica* for further development of biofuels and natural products.

**Electronic supplementary material:**

The online version of this article (doi:10.1186/s13068-016-0687-7) contains supplementary material, which is available to authorized users.

## Background

Within a single fungal species, chromosome number and content can vary widely [1]. Genomic instability has been documented in chromosomal changes in yeasts [2], induced in *Candida albicans* by centromere removal [3], and observed in the fragmentary gene order conservation of filamentous Ascomycetes [4]. *Yarrowia lipolytica* also has evidence of inter-strain differences, demonstrated by CHEF gel [5]. Studies in *Y. lipolytica* have primarily been done in three popular genetic backgrounds: W29 (Wild-type French strain ATCC20460™), H222 (wild-type German strain), and CBS6142-2 (the wild-type American strain) [6]. The Po1 series, derived of a set of backcrosses between W29 and CBS6142-2 [7], have been used for a number of studies. CLIB122, or E150, the reference genome sequence, is derived of W29 in a cross with YB423-12, isolated from milled corn fiber tailings [8]. Genome sequencing efforts have covered some original isolates and additional progeny from genetic studies, including strain W29 [9], and one of a backcrossed series, Po1f [10]. Molecular genetic tools in Yarrowia include ablation of the *ku70* ortholog, done in the citric acid producer H222, and in Po1d, which increases the rate of homologous recombination during transformation [11, 12]. Further genome sequencing is needed, as included for Po1g below, to clarify gene content and regulatory region differences between *Y. lipolytica* strains.

*Yarrowia lipolytica* has a precedence of organelle studies, particularly for peroxisome biogenesis and dynamics, including six stages of microbody development with differing size and contents [13]. Study of catabolism in the peroxisome, and by lipases [14], modeling efforts [15, 16], and perturbation of both beta-oxidation and elements of the lipid biosynthetic pathways [17] have contributed to engineering desired products such as carotenoids [18], and omega-3 fatty acids [19]. The availability of tools to identify organelle compartments would facilitate studies of this type.

A variety of stains are available for visualizing different intracellular compartments in yeast. FUN-1, Nile Red, MitoTracker, ER-Tracker, and DAPI among others can be used to visualize the vacuole, lipid droplet, mitochondria, endoplasmic reticulum, and nucleus, respectively. Compendia of cell staining techniques are available for specific organisms [20] or specific organelles [21, 22]. However, in *Y. lipolytica*, we observed poor penetration, toxicity, the need for fixation, and resistance to stain uptake, consistent with many fungi [23–26]. Fixation frequently alters intracellular morphology and staining effectiveness when using these stains. Similarly, changes in growth conditions, or incubation buffer may perturb stain application, as stain efflux differences can be pH dependent [27]. The cell wall changes thickness in an age- and nutrient-dependent manner [28], and may provide an additional barrier to permeability. Probe or stain molecule size requiring a chemical vehicle which increases permeability of cell membranes (e.g., DMSO) may also affect cell health and cogency of data, just as complications of dye delivery under nutrient specific conditions and removal of excess dye may perturb cell integrity. Organelle-specific proteins tagged with green fluorescent protein (GFP) overcome many of these limitations, particularly for life cell imaging.

The technique of using GFP labeled proteins for localization and expression is more than two decades old [29]. The Yarrowia community has developed plasmids for over-expression, or carbon-induced expression using a non-integrating cassette for secretion or exogenous protein expression [7, 30, 31]. Their use is dependent upon having the correct auxotrophs and efficient transformation methods [17, 32]. Fluorescent protein studies in *Y. lipolytica* have encompassed tagging for localization of Fat1p, Fat4p, and Faa1p using plasmids [33, 34]. Similarly, hybrid promoter studies have utilized fluorescent proteins [35], and transcription factors have been localized using a GFP-fusion expressed from a plasmid [36]. However, sets of *Y. lipolytica* strains with GFP tagged organelles are not available.

Tools presented here will allow definition of pathways, localization of biosynthetic enzymes, and organelle dynamics in living cells. We developed an isogenic *ku70::hph* strain set for improved homologous recombination efficiency when transforming PCR products, and assessing localization of proteins within a cell by fluorescent tagging under a high expression promoter using auxotrophic selection of transformants or integrants. This genetic background was sequenced and annotated to facilitate genetic studies. A superfolder GFP gene which shows bright fluorescence [37], was codon optimized for multi-modal use in *Y. lipolytica*, demonstrated by an enzyme over-expression collection using lipid biosynthesis proteins. To query organelle dynamics in response to changing environmental conditions and build a tool set for co-localization studies, we built the *Y. lipolytica* Cell Atlas composed of seven strains with different cell compartment labels in both auxotrophic and prototrophic backgrounds. This work provides a consistent set of strains and tools for genetics and cell biology in *Y. lipolytica* and demonstrates the dynamic nature of organelles important for energy metabolism under conditions relevant to industrial biofuel production.

## Results and discussion

### Construction of isogenic NHEJ-deficient auxotrophic strains

**Fig. 1:**
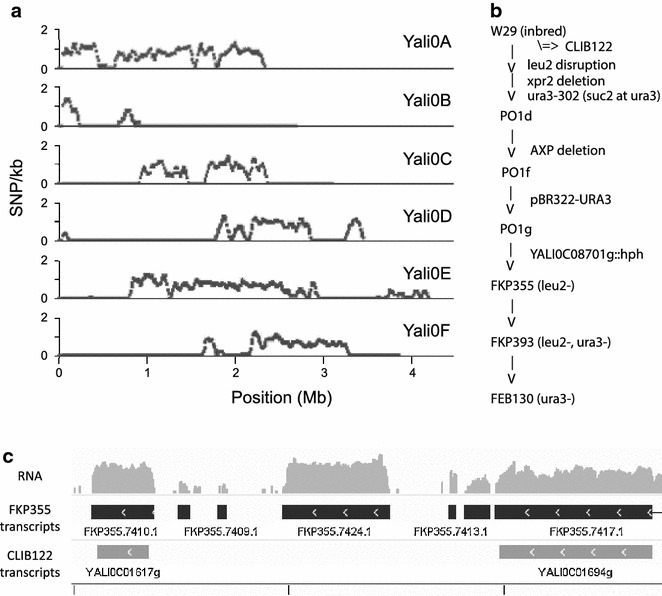
FKP355 genomic background and identified transcripts. **a** SNP frequency map of FKP355 versus CLIB122 genome shows genomic regions that differ from the available reference. Chromosome A is almost wholly from another background, while other chromosomes show distinct blocks of variation. **b** Heritage of the strains generated in this work derived from the widely used Po1 series of progeny in an inbred W29 background. **c** New transcripts undiscovered in the reference CLIB122 strain have been annotated in FKP355 by RNA sequencing. See Additional file 2 for a table of annotations in FKP355

Previous work has shown that a deletion of the *ku70* ortholog increases transformation efficiency and rate of recovery of transformants targeted to specific loci [11, 12]. This removes a non-homologous DNA repair process which allows random integration of DNA and so decreases mis-localization of constructs intended for a particular locus. The *Y. lipolytica ku70* ortholog was identified as YALI0C08701g by BLAST. We set out to construct a set of isogenic strains in which *ku70* was replaced with a gene conferring hygromycin resistance (hygromycin phosphotransferase; *hph*) in the Po1g genetic background, commonly used for studies in fungal biotechnology [7, 30] (Table 1). The majority of selective markers available in *Y. lipolytica* are amino acid and nucleobase auxotrophies. Hygromycin B, derived from *Streptomyces hygroscopicus,* is a translation inhibitor effective in other fungal systems. It has been demonstrated to be useful in *Y. lipolytica* in a loxP Cre-removable system [11, 38]. Hygromycin B is an archival and successful selective agent for ascomycetes and hemiascomycetes [39–42]. The wild-type strains ATCC20460™ and ATCC18944 ™ as well as Po1g have different natural resistance levels to the antibiotic Hygromycin B (see Additional file 1: Figure S1). Efficient expression of *hph* is key for use as a selective marker. We therefore codon optimized *hph* to enable stricter selection. In order to produce auxotrophic markers *leu2* and *ura3* separately and together, we replaced *ku70* (YALI0C08701g) with *hph* driven by the *tef* promoter in the *leu2* auxotroph Po1g as a starting point to make FKP355 (*matA*, *ku70*::*hph*+, *leu2*-*270*) (Fig. 1b). Successful replacement was confirmed by Southern blot. Figure S2E (see Additional file 1) shows the proper length of all genomic DNA fragments after digestion with BglII, EcoRV, or PvuII restriction endonucleases. We then complemented *leu2*-*270* by replacement with *leu2*+ to make FKP391 (*matA*, *ku70*::*hph*+, *leu2*-*270*::*leu2*+), providing a prototroph. To generate a double auxotroph from leucine-requiring FKP355, we selected for disruption of *ura3* by selection on 5-FOA containing medium to make FKP393 (*matA*, *ku70*::*hph*+, *leu2*-*270*, *ura3* ^−^). The complicated *ura3* locus in FKP355 contains *ura3::suc2*+ adjacent to an intact copy of *ura3*. The disrupted and intact copies of *ura3* recombined leaving only *ura3::suc2*+ in FKP393. Analysis of RNA suggests that the plasmid sequence between the disrupted and intact copies of *ura3* was lost, indicative of a loop out event while genes adjacent to *ura3* (YALI0E26653g and YALI0E26785g) are intact (RNA data not shown). We then complemented *leu2*-*270* in FKP393 by replacement with *leu2*+ to make FEB130 (*matA*, *ku70*::*hph*+, *ura3* ^−^) using the gene from wild-type strain W29 (ATCC 20460™) [9]. These strains represent a complimented prototroph and a set of auxotrophs in an isogenic NHEJ-deficient background for biological investigation (Fig. 1b).

**Table 1:** Strains used in this study

Strain	Genotype	Reference
W29	ATCC20460	Gaillardin et al. [137]
P01g	*matA, xpr2*-*332, axp*-*2, leu2*-*270*	Madzak et al. [7]
FKP355	*matA, xpr2*-*332, axp*-*2, ku70::hph*+*, leu2*-*270*	This work
FKP391	*matA, xpr2*-*332, axp*-*2, ku70::hph*+*, leu2*-*270::leu2*+	This work
FKP393	*matA, xpr2*-*332, axp*-*2, ku70::hph*+*, leu2*-*270, ura3*	This work
FEB130	*matA, xpr2*-*332, axp*-*2, ku70::hph*+*, leu2*-*270::leu2*+*, ura3*	This work
FEB56	*matA, xpr2*-*332, axp*-*2, ku70::hph*+*, leu2*-*270, erg6*-*sfGFP:leu2*+	This work
FEB64	*matA, xpr2*-*332, axp*-*2, ku70::hph*+*, leu2*-*270, pex13*-*sfGFP:leu2*+	This work
FEB91	*matA, xpr2*-*332, axp*-*2, ku70::hph*+*, leu2*-*270, hH1*-*sfGFP:leu2*+	This work
FEB92	*matA, xpr2*-*332, axp*-*2, ku70::hph*+*, leu2*-*270, rdl2*-*sfGFP:leu2*+	This work
FEB93	*matA, xpr2*-*332, axp*-*2, ku70::hph*+*, leu2*-*270, aim17*-*sfGFP:leu2*+	This work
FEB94	*matA, xpr2*-*332, axp*-*2, ku70::hph*+*, leu2*-*270, emc2*-*sfGFP:leu2*+	This work
FEB96	*matA, xpr2*-*332, axp*-*2, ku70::hph*+*, leu2*-*270, cpy1*-*sfGFP:leu2*+	This work
FEB97	*matA, xpr2*-*332, axp*-*2, ku70::hph*+*, leu2*-*270, arx1*-*sfGFP:leu2*+	This work
FEB103	*matA, xpr2*-*332, axp*-*2, ku70::hph*+*, leu2*-*270, vrg4*-*sfGFP:leu2*+	This work
FEB100	*matA, xpr2*-*332, axp*-*2, ku70::hph*+*, leu2*-*270, erg6*-*sfGFP:leu2*+*, ura3*	This work
FEB98	*matA, xpr2*-*332, axp*-*2, ku70::hph*+*, leu2*-*270, pex13*-*sfGFP:leu2*+*, ura3*	This work
FEB87	*matA, xpr2*-*332, axp*-*2, ku70::hph*+*, leu2*-*270, hH1*-*sfGFP:leu2*+*, ura3*	This work
FEB83	*matA, xpr2*-*332, axp*-*2, ku70::hph*+*, leu2*-*270, rdl2*-*sfGFP:leu2*+*, ura3*	This work
FEB84	*matA, xpr2*-*332, axp*-*2, ku70::hph*+*, leu2*-*270, aim17*-*sfGFP:leu2*+*, ura3*	This work
FEB89	*matA, xpr2*-*332, axp*-*2, ku70::hph*+*, leu2*-*270, emc2*-*sfGFP:leu2*+*, ura3*	This work
FEB85	*matA, xpr2*-*332, axp*-*2, ku70::hph*+*, leu2*-*270, cpy1*-*sfGFP:leu2*+*, ura3*	This work
FEB86	*matA, xpr2*-*332, axp*-*2, ku70::hph*+*, leu2*-*270, arx1*-*sfGFP:leu2*+*, ura3*	This work
FEB90	*matA, xpr2*-*332, axp*-*2, ku70::hph*+*, leu2*-*270, vrg4*-*sfGFP:leu2*+*, ura3*	This work

### Genome sequencing, assembly, and annotation of NHEJ-deficient base strain

*Yarrowia lipolytica* strains from the Po1 series of auxotrophs [7, 31, 43] are a commonly used genetic background for experimental studies. However, the available CLIB122 reference genome was produced from an outcrossed strain such that approximately half the reference genome is representative of the sequence of the Po1 series. Recently, the Po1f strain was sequenced [10], and we have sequenced strains derived from W29 [9] to shed additional light on this genetic background. In this study, we sequenced the genome of the *ku70*::*hph* strain (FKP355, *ku70* mutant of Po1g) from which all the strains described herein are derived. We assembled 680 contains from 23,305,816 paired-end 150 nucleotide reads. The resulting assembly has a size of 20.3 Mb (*N*50 162,550 bp, *N*Max 562,182 bp, median coverage 45×) and GC content of 48.2%. By analyzing SNPs, we identified which regions in this strain are derived from a genetic background distinct from the CLIB122 reference genome based on SNP frequency. We identified few conserved SNPs (0.0034 ± 0.0075 SNP/kb) in regions of CLIB122 derived from W29 while divergent regions have 0.916 ± 0.483 SNP/kb (Fig. 1a). Comparison of CLIB122 gene models to the FKP355 assembly identified 26 genes with low (less than 10%) homology to FKP355. Sixteen of these are transposable elements and six are ORFs without known functional domains or homology to any other species, suggesting they may be pseudogenes. Two belong to the *matb* mating-type locus of CLIB122 and would not be expected in FKP355 which is *mata*. We did not identify *ku70* since it was replaced with *hph* in FKP355, leaving the predicted acid phosphatase Yali0E35222g as the only gene likely to be physiologically relevant that is present in CLIB122 but not FKP355. We identified 8423 transcripts based on alignment of RNA-sequencing data [15] from which 8571 proteins were predicted and annotated (Additional file 2: Table S1–Protein annotations). Among the 8571 annotations, there are examples of proteins with RNA-seq coverage [15] which do not have a reciprocal best BLAST hit in the CLIB122 genome (Fig. 1c). Thirty-seven transcripts with low homology to the CLIB122 reference genome [44], that contain long open reading frames, were identified as potentially novel protein coding genes in FKP355 (Table 2, New proteins). Most of the novel proteins are predicted from transcripts that are clustered in particular genomic regions suggesting that small stretches of DNA containing multiple genes are not present or were not assembled in strain CLIB122. Within these regions are a variety of important enzymes that are not found in the CLIB122 genome including homogentisate dioxygenase, fumarylacetoacetase, and argininosuccinate synthase. Also present within the new proteins are those belonging to multi-gene families with low homology to species other than *Y. lipolytica*. Within these multi-gene families are an uncharacterized group of five general substrate transporters, and a group of six cytochrome p450 enzymes that may endow *Y. lipolytica* with, as yet undocumented, metabolic capabilities. The *ura3* gene is known to have a large indel in the CLIB122 strain, rendering it a uracil auxotroph, but has been reintroduced into the FKP355 genetic background. We identified the transcript with the coding gene for Ura3p in this list as a demonstration of its utility.

**Table 2:** New transcripts by RNA-seq in FKP355 compared to CLIB122

Protein	FKP355 contig	Start	Stop	Annotation	Transcript ID to CLIB122 (%)	Multigene families
FKP355.266.1|m.2906	NODE_1012	5782	7332	–	65.4	
FKP355.702.1|m.5424	NODE_1296	70375	70970	–	85.7	
FKP355.831.1|m.6293	NODE_139	1	1282	–	0.0	3
FKP355.1249.1|m.9149	NODE_187	5	419	–	0.0	
FKP355.1708.1|m.12810	NODE_215	3	799	–	10.3	4
FKP355.1712.1|m.12805	NODE_215	968	1934	Metal dependant phosphohydrolase	0.0	2
FKP355.1717.1|m.12793	NODE_215	1995	2977	Ribosomal protein L15	0.0	
FKP355.1713.1|m.12814	NODE_215	3373	5348	Exonuclease domain containing protein	0.0	4
FKP355.1716.1|m.12796	NODE_215	6426	8721	LDB19 protein	0.0	
FKP355.2293.1|m.17448	NODE_239	75445	81675	Retrotransposon ty3-gypsy subclass	8.8	
FKP355.2453.1|m.18283	NODE_243	3	6139	Orotidine 5-phosphate decarboxylase (ura3)	15.9	
FKP355.2489.1|m.18598	NODE_263	3977	4883	Kelch repeat protein	0.0	10
FKP355.4404.1|m.32419	NODE_388	4	178	–	0.0	
FKP355.5225.1|m.38433	NODE_526	112122	112302	–	84.0	
FKP355.5430.1|m.40180	NODE_58	3	1702	Homogentisate dioxygenase	5.4	
FKP355.5431.1|m.39976	NODE_58	2102	5597	Fumarylacetoacetase	0.0	
FKP355.5453.1|m.40112	NODE_58	7439	8998	Glycosyltransferase	0.0	
FKP355.5436.1|m.40252	NODE_58	9224	10476	Clathrin coat assembly protein	0.0	
FKP355.5440.1|m.40006	NODE_58	10559	14552	Conserved hypothetical protein	0.0	
FKP355.5440.1|m.40007	NODE_58	10559	14552	Conserved hypothetical protein	0.0	
FKP355.5469.1|m.39839	NODE_58	15038	16629	Argininosuccinate synthase	0.0	
FKP355.5446.1|m.39944	NODE_58	16715	18168	Serine threonine protein phosphatase	0.0	
FKP355.5441.1|m.39812	NODE_58	18861	21039	–	0.0	9
FKP355.5445.1|m.39907	NODE_58	22161	25000	Protein with serine active lipase domain	0.0	
FKP355.5400.1|m.39579	NODE_581	1309	3097	Alcohol dehydrogenase	0.0	2
FKP355.5427.1|m.39610	NODE_587	60501	62936	Mfs general substrate transporter	17.2	5
FKP355.6195.1|m.45807	NODE_6	14226	14433	Hsp70-like protein	70.7	5
FKP355.6384.1|m.47130	NODE_740	6	1175	–	16.5	
FKP355.6380.1|m.47125	NODE_740	1269	1795	–	0.0	
FKP355.6381.1|m.47133	NODE_740	2062	2362	–	0.0	4
FKP355.6855.1|m.50863	NODE_797	49605	50628	Putative cation transporter	6.5	
FKP355.6832.1|m.50956	NODE_797	50984	52853	Cytochrome p450 alkane	0.0	6
FKP355.6828.1|m.50886	NODE_797	53013	54512	–	0.0	
FKP355.7207.1|m.53424	NODE_845	428661	429387	–	61.9	8
FKP355.7712.1|m.57224	NODE_87	2	1702	–	0.0	6
FKP355.7709.1|m.57217	NODE_872	12	192	–	0.0	
FKP355.7976.1|m.59101	NODE_94	816	1913	–	0.0	2

### Construction of a multipurpose vector for rapid expression of fluorescently tagged proteins in *Yarrowia lipolytica*

**Fig. 2:**
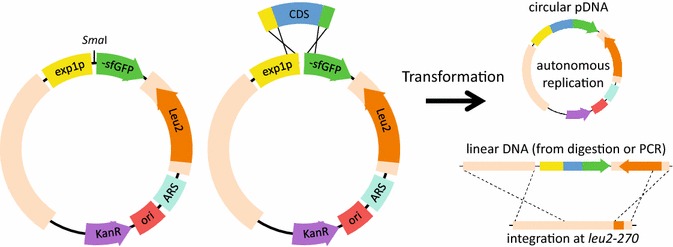
Utilization of vector pYL15 for expression of sfGFP fusion proteins. Vector pYL15 is linearized by digestion with *Sma*I. Coding sequences of interest are amplified with primers containing short 5′ and 3′ overhangs homologous to the pYL15 cut site. The vector and coding sequence are assembled and transformed into *E. coli*. The pYL15 vector contains *Y. lipolytica* centromere and origin of replication sequences derived from ARS68 [46] as well as *leu2* for selection. Map is not drawn to scale to accommodate text

We constructed a multipurpose expression vector pYL15 (GenBank accession: KU378202) containing a codon optimized version of the superfolder GFP gene (sfGFP) [37] preceded by a 10× glycine linker and driven by the high expression *exp1* promoter [35] to enable rapid analysis of protein localization by expression of GFP fusion proteins in *Y. lipolytica*. Coding sequences of interest are amplified by PCR from genomic or complementary DNA with primers containing short overhangs homologous to either side of a SmaI site present in the pYL15 vector, which contains the full length *leu2* gene from *Y. lipolytica*. The vector and coding sequence are then assembled by Gibson assembly [45] to reduce the chances of producing vector without an insert (Fig. 2). The vector contains centromere and origin of replication sequences from ARS68 [46] enabling autonomous replication when transformed as a circular plasmid but when linear integrates at the *leu2*-*270* locus present in strains commonly used for *Y. lipolytica* genetic studies [7, 43] (Fig. 2). Transformed cells are typically grown in yeast nitrogen base medium with ammonium sulfate (YNB) requiring biosynthesis of leucine to select for maintenance of the plasmid. This system enables rapid assembly and high-level expression of sfGFP-tagged proteins in *Y. lipolytica*. The ability to clone any coding sequence without the use of restriction enzymes and using a single linear plasmid makes this construct amenable to moderate- and high-throughput protein localization studies. To demonstrate the usefulness of pYL15, we cloned enzymes involved in triglyceride biosynthesis and expressed them extra-chromosomally with sfGFP tags to determine their localization.

### Expression of sfGFP-tagged lipid biosynthetic enzymes reveals their localization

**Fig. 3:**
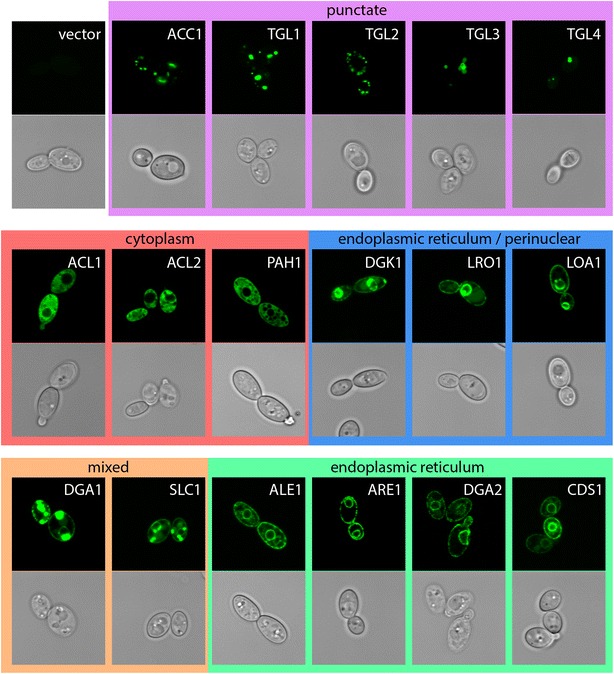
Localization patterns of lipid biosynthetic enzymes in *Y. lipolytica*. Coding sequences for lipid biosynthetic enzymes were cloned into plasmid pYL15 to create C-terminal fusion proteins with sfGFP. Plasmids were transformed into strain FKP355 and green fluorescence visualized by laser scanning confocal microscopy

**Fig. 4:**
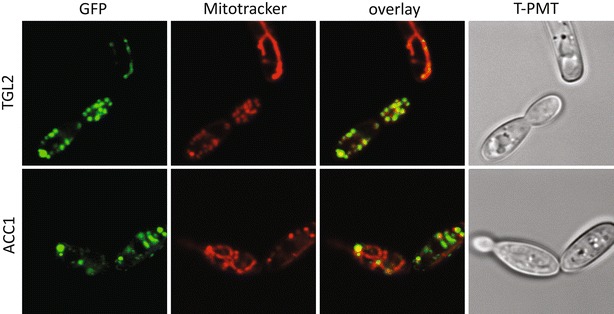
Mitochondria localized enzymes. *Y. lipolytica* cultures expressing sfGFP fusion proteins with a punctate pattern were grown to late log phase in YNB, fixed briefly with formaldehyde, and stained with MitoTracker deep red to visualize mitochondria. Co-localization with green fluorescent proteins was assessed by laser scanning confocal microscopy

**Fig. 5:**
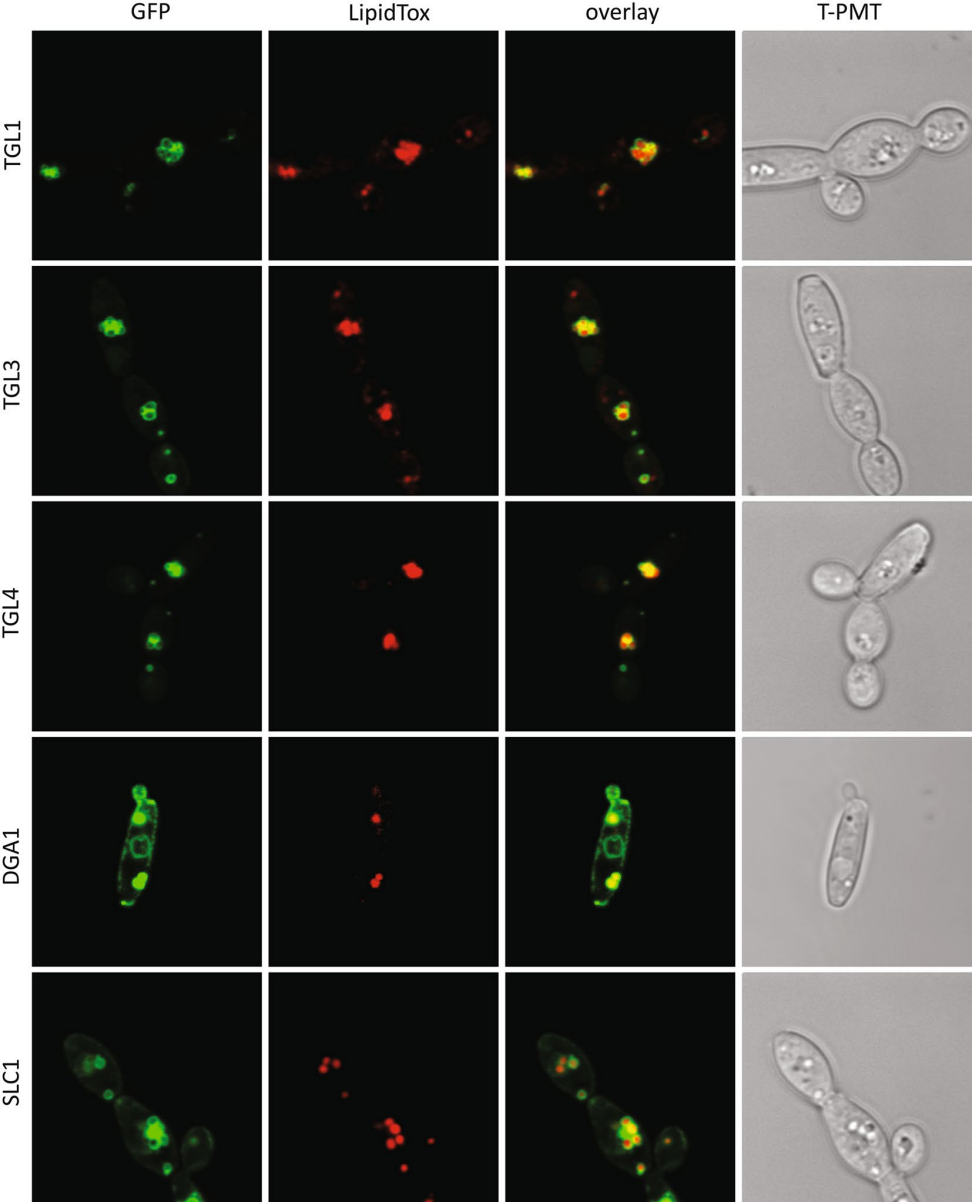
Lipid droplet localized enzymes. *Y. lipolytica* cultures expressing sfGFP fusion proteins with a punctate pattern were grown to late log phase in YNB, fixed briefly with formaldehyde, and stained with LipidTOX *red* to visualize lipid droplets. Co-localization with *green* fluorescent proteins was assessed by laser scanning confocal microscopy

*Yarrowia lipolytica* is a model organism for the study of metabolism in the context of lipid production for biofuels and other value added lipid products. A number of compartmentalized metabolic models have been constructed for *Y. lipolytica* that incorporate data derived primarily from homologous proteins in other organisms [15, 16, 47]. We therefore assessed the localization of enzymes involved in triglyceride biosynthesis from glucose in *Y. lipolytica* to determine where key metabolic reactions occur and to improve on location-dependent metabolic models in *Y. lipolytica* (Fig. 3; Table 3). The initial building block for fatty acids is Acetyl-CoA, which is produced from citrate by ATP-ctirate lyase (ACL). The presence of ACL in *Y. lipolytica* is a major distinction between its metabolic network and that of non-oleaginous organisms [48] and deletion results in reduced ability to accumulate lipids [49]. Both subunits of this enzyme localize to the cytosol where they presumably interact to drive lipid accumulation from citrate. Conversion of Acetyl-CoA to Malonyl-CoA by Acetyl-CoA Carboxylase (ACC) is the committed step in fatty acid production and has been successfully used to produce higher quantities of lipids in *Y. lipolytica* [50, 51]. In *Saccharomyces cerevisiae*, ACC is expressed by two genes, *acc1* and *hfa1*, which encode cytosol- [52], and mitochondria-localized [53] variants. *Y. lipolytica* has a single copy of *acc1* with a putative mitochondrial targeting signal that exhibits punctate localization that partially overlaps with mitochondria (Figs. 3, 4). Acetyl-CoA and Malonyl-CoA are then utilized by the Type I Fatty Acid Synthase complex, which in *S. cerevisiae* consists of six copies each of cytoplasmically located Fas1p and Fas2p [54, 55] and Beta-ketoacyl-ACP synthase to make acyl-CoA molecules which primarily consist of 16:0, 18:1, 18:2 chains when grown on glucose [28, 56]. The genes encoding homologs of *fas1* and *fas2* in *Y. lipolytica* are both predicted to have short introns. Intriguingly, the splice junction at the 5′ end of the intron is within the translation start site of *fas2* and directly adjacent to the translation start site of *fas1*, respectively, potentially enabling splicing as a strong regulator of expression for these genes. We were unable to detect a green fluorescent protein signal in *fas1*-*sfGFP* or *fas2*-*sfGFP* strains constructed with or without the short leader introns (data not shown) suggesting that translation of *fas1* and *fas2* may be tightly controlled by an as yet unknown mechanism in *Y. lipolytica*. Acyl-CoA molecules produced by the fatty acid synthase complex are utilized by enzymes during lipid biosynthesis. The initial acyl group is attached to the lipid backbone by the glycerol-3-phosphate sn-1 acyltransferase *sct1*, which we were unable to detect green fluorescent protein signal from. A second acyl chain can be added to lyso-phosphatidic acid by a number of different enzymes in *S. cerevisiae* including Slc1p [57], Ale1p [58, 59], and Loa1p [60], to produce phosphatidic acid. Interestingly, we found that Slc1-sfGFPp localizes to both the endoplasmic reticulum and the periphery of lipid droplets while Ale1-sfGFPp and Loa1-sfGFPp localize exclusively to the endoplasmic reticulum, consistent with results in *S. cerevisiae* [61, 62] (Figs. 3, 5). Utilization of phosphatidic acid represents a major regulatory point in lipid homeostasis (for review see: [62–64]). CDP-Diacylglycerol Synthase (CDS) activates phosphatidic acid by addition of CDP prior to release of CMP during the construction of the polar head groups for phospholipid synthesis [65], localized in the ER in *Y. lipolytica* (Fig. 3). Alternatively, phosphatidic acid is dephosphorylated to form diacylglycerol by Phosphatidate Phosphatase. In *S. cerevisiae*, the Phosphatidate Phosphatase Pah1p is regulated by phosphorylation to maintain lipid homeostasis between storage and phospholipids [66–69], and the homolog of Pah1p in *Y. lipolytica* resides in the cytosol (Fig. 3). The endoplasmic reticulum localized enzyme Diacylglycerol Kinase (Dgk1-sfGFPp) phosphorylates diacylglycerol to produce phosphatidic acid (Fig. 3). Alternately, acylation of diacylglycerol to produce storage lipids in the form of triglycerides is performed by Dga1p, Dga2p, and Lro1p. Dga1p and Dga2p utilize acyl-CoA as the acyl chain donor while the lecithin cholesterol acyltransferase Lro1p utilizes phospholipids [70–72]. Lro1-sfGFPp localizes to the endoplasmic reticulum and like Loa1-sfGFPp and Dgk1-sfGFPp is found primarily at the nuclear periphery (Fig. 3). In contrast, Dga1-sfGFPp localizes to the endoplasmic reticulum and the periphery of lipid droplets (Figs. 3, 5), suggesting it is the major producer of lipid droplet stored triglycerides in *Y. lipolytica*, while Dga2-sfGFPp localizes exclusively to the endoplasmic reticulum (Fig. 3). Acyl-CoA is also used for the esterification of sterols by Are1p [73] which localizes to the endoplasmic reticulum in *Y. lipolytica* (Fig. 3). We are also interested in the localization of enzymes that utilize triglycerides. *Y. lipolytica* has four triglyceride lipases that catalyze the cleavage of triglycerides to produce free fatty acids. We found that three of them localize to lipid droplets (Tgl1-sfGFPp, Tgl3-sfGFPp, and Tgl4-sfGFPp; Fig. 5) in a manner consistent with previous findings for Tgl3p and Tgl4p [74], while Tgl2-sfGFPp localizes to the mitochondria (Figs. 3, 4) as in *S. cerevisiae* [75].

**Table 3:** Localization of Yarrowia and Saccharomyces lipid biosynthetic enzymes

*Y. lipolytica gene*	*S. cerevisiae* homolog	sfGFP tag	*S. cerevisiae*
YALI0C11407g (*acc1*)	*acc1, hfa1*	MI	CY, MI
YALI0E34793g (*acl1*)	–	CY	–
YALI0D24431g (*acl2*)	–	CY	–
YALI0F19514g (*ale1*)	*ale1*	ER	ER
YALI0F06578g (*are1*)	*are1*	ER	ER
YALI0D07986g (*dga2*)	*are2*	ER	ER
YALI0E14443g (*cds1*)	*cds1*	ER	ER, MI
YALI0E32769g (*dga1*)	*dga1*	ER, LD	ER, LD
YALI0F19052g (*dgk1*)	*dgk1*	ER	ER
YALI0C14014g (*loa1*)	*loa1*	ER	ER, LD
YALI0E16797g (*lro1*)	*lro1*	ER	ER
YALI0D27016g (*pah1*)	*pah1*	CY	CY, ER
YALI0E18964g (*slc1*)	*slc1*	ER, LD	ER, LD
YALI0E32035g (*tgl1*)	*tgl1*	LD	ER, LD
YALI0E31515g (*tgl2*)	*tgl2*	MI	MI
YALI0D17534g (*tgl3*)	*tgl3*	LD	LD
YALI0F10010g (*tgl4*)	*tgl4*	LD	LD

**Fig. 6:**
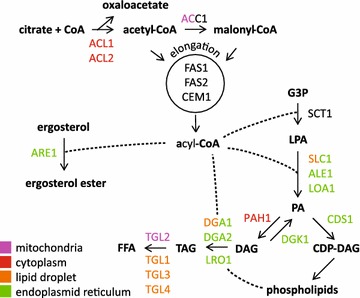
Lipid biosynthesis in *Y. lipolytica*. The localization of enzymes involved in production of lipids from citrate is shown along with results from our analysis of localization. Enzyme names with multiple colors indicate localization to more than one compartment. *ACL* ATPcitrate lyase, *ACC* Acetyl-CoA carboxylase, *ALE1* 1-acyl-sn-glycerol-3-P acyltransferase, *ARE* Acyl-CoA:sterol acyltransferase, *CDS* CDP-diglyceride synthase, *CEM1* β-ketoacyl synthase, *DGA* Diacylglycerol acyltransferase, *DGK* Diacylglycerol kinase, *FAS* Fatty acid synthase, *LOA1* 1-acyl-sn-glycerol-3-P acyltransferase, *LRO1* Phospholipid:diacylglycerol acyltransferase, *PAH1* Phosphatidate phosphatase, *SLC1* 1-acyl-sn-glycerol-3-P acyltransferase, *SCT1* Glycerol-3-P o-acyltransferase, *TGL* Triacylglycerol lipase. *G3P* glycerol-3-phosphate, *LPA* lyso-phosphatidic acid, *PA* phosphatidic acid, *DAG* diacylglycerol, *TAG* triacylglycerol, *FFA* free fatty acid

The locations we determined are frequently consistent with homologs of enzymes in *S. cerevisiae* [61, 62] with all five of the exceptions being enzymes where we identified only a single location in *Y. lipolytica* but for which multiple locations have been determined in *S. cerevisiae* (Fig. 3). Additional experimentation in *Y. lipolytica* is likely to augment these additional locations. For example, we determined that Pah1-sfGFPp is localized to the cytosol; however, its substrate, phosphatidic acid, resides within the endoplasmic reticulum membrane suggesting that Pah1p must interact at least transiently with the endoplasmic reticulum. We found that many of the lipid biosynthesis enzymes localize to the endoplasmic reticulum; however, GFP data alone cannot determine ER lumen or cytosol direction of enzyme activity. In summary, we identified the location of 17 enzymes involved in lipid metabolism in *Y. lipolytica* by plasmid-based expression, many of which localize to multiple organelles (Fig. 6). These data provide evidence for additional locations of Dga1p and Slc1p and previously undetermined locations for Dga2p and Loa1p that can be used to improve compartmentalized metabolic models for *Y. lipolytica* [15].

### Construction of an atlas of endogenous sfGFP-tagged organelles

**Fig. 7:**
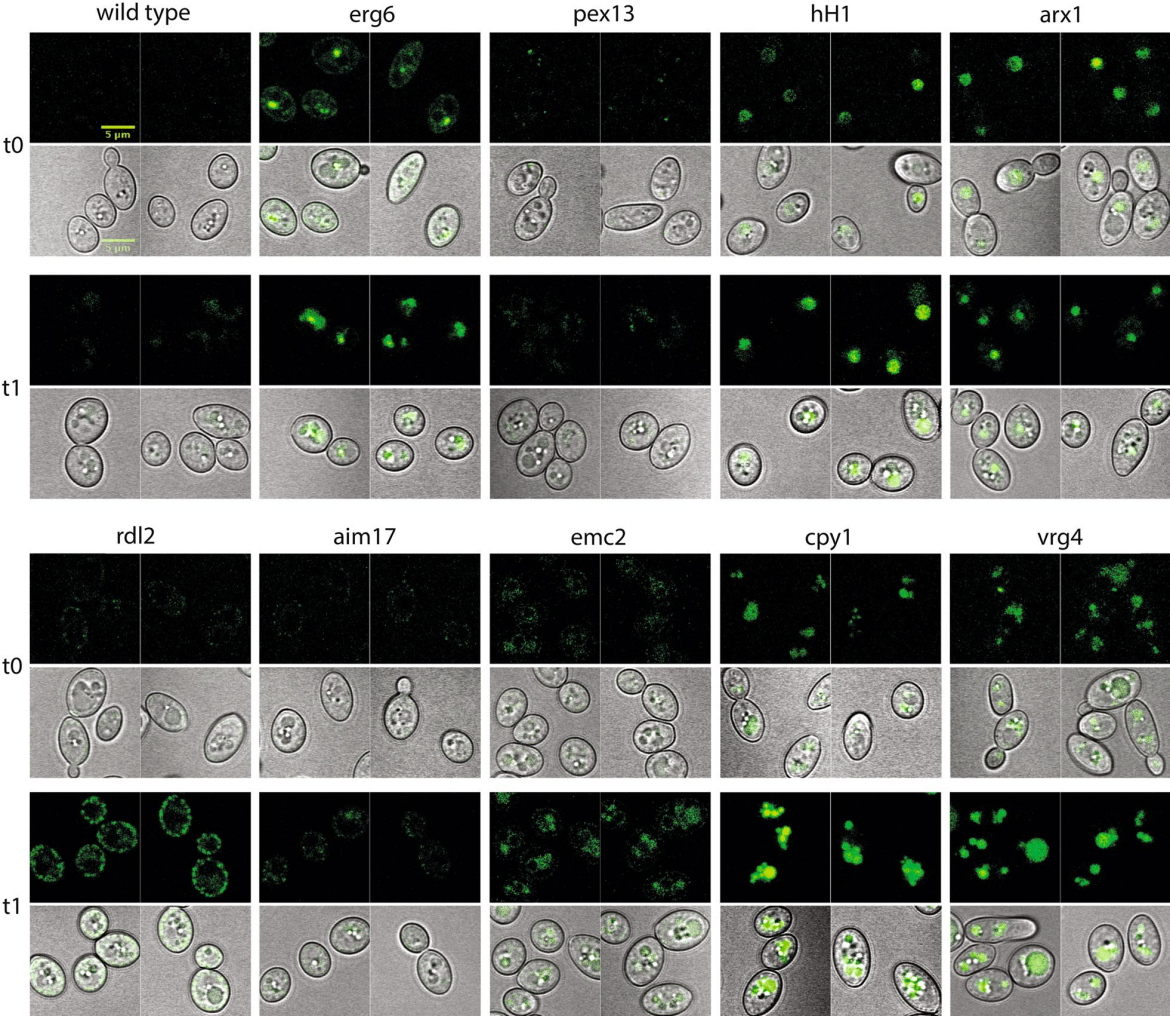
Cell atlas prototrophs. The strains labeling 7 organelle compartments within cells in a prototrophic background. FEB130 was transformed with an endogenously targeted PCR products containing fragments to C-terminally tag organelle-specific proteins followed by a complimenting URA3 gene. Increased lipid droplet accumulation is accompanied by loss of peroxisomes, increase in hH1 signal, and other cellular changes tracked in a live cell without further processing steps. Strains were imaged at 16 h in YPD (t0) and after 20 h in Y-D media (t1). *Top* GFP channel; *bottom* GFP with transmission PMT overlay

**Fig. 8:**
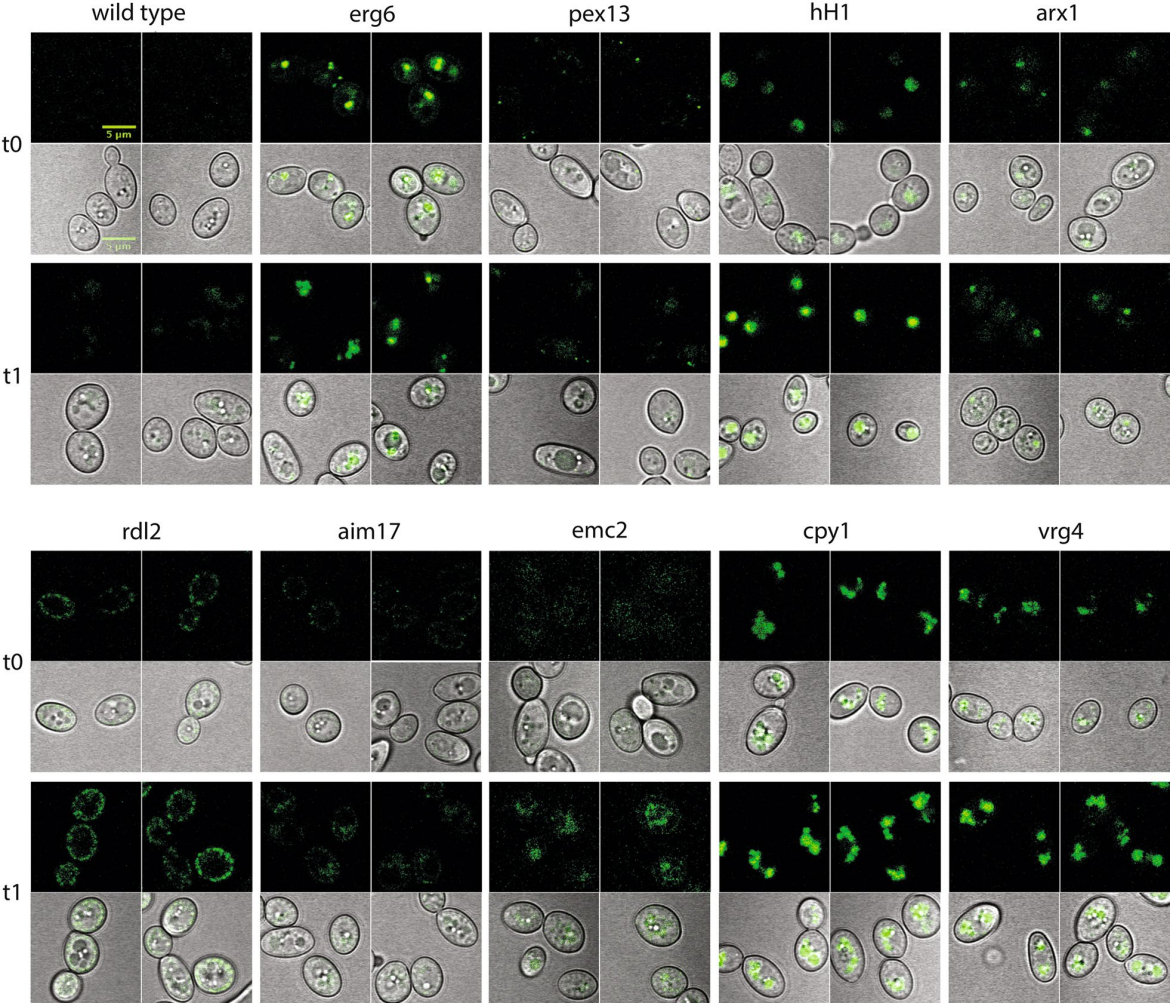
Cell atlas auxotrophs. An atlas showing cell compartment-specific proteins at 16 h in YPD (t0) and after 20 h of nitrogen limitation in Y-D media, lacking a nitrogen source (t1). Localization is the same as in the prototrophs, leaving open a *ura3* marker for further strain construction. *Top* GFP channel; *bottom* GFP with transmission PMT overlay

To overcome limitations of organelle identification by staining and fixation, we generated an atlas of strains with green fluorescent organelles by tagging genes with sfGFP at their endogenous locus (Figs. 7, 8). These intracellular markers respond to native promoter control, rather than being constitutively expressed from plasmids, and avoid overexpression toxicity, which has been suggested in other eukaryotic systems [76]. We chose non-essential proteins which show high and consistent expression, membrane association to define the organelle, DNA or rRNA association for the nucleus, and successful tagging in the *S. cerevisiae* GFP protein libraries [77, 78]. We identified genes encoding *Y. lipolytica* homologs of proteins known to be specific to particular organelles in *S. cerevisiae* including proteins that localize to the nucleus (*hH1*, YALI0B16280g; *arx1*, YALI0C05599g), mitochondrion (*rdl2*, YALI0F29667g; *aim17*, YALI0F16357g), peroxisome (*pex13*, YALI0C05775g), lipid droplet (*erg6*, YALI0F08701g), endoplasmic reticulum (*emc2*, YALI023188g), vacuole (*cpy1*, YALI0A18810g), and golgi apparatus (*vrg4*, YALI0F21791g) (Table 4). We constructed the green fluorescent organelle atlas in a prototrophic and auxotrophic (*ura3*−) *ku70*::*hph* genetic background to enable observation of experimental perturbations in mutant and wild-type–sfGFP strains, and to provide a convenient tool for co-localization studies.

**Table 4:** Yarrowia identity and coverage compared to S288C proteins

Name	*Y. lipolytica*	*S.cerevisiae*	Coverage (%)	Identity (%)
hH1	YALI0B16280g	YPL127C	59	54
arx1	YALI0C05599g	YDR101C	90	33
cpy/prc1	YALI0A18810g	YMR297W	83	66
vrg4	YALI0F21791p	YGL225W	94	61
aim17	YALI0F16357p	YHL021C	89	28
rdl2	YALI0F29667g	YOR286W	67	49
emc2	YALI0C23188g	YJR088C	92	25
pex13	YALI0C05775g	YLR191W	79	50
erg6	YALI0F08701g	YML008C	94	65

**Fig. 9:**
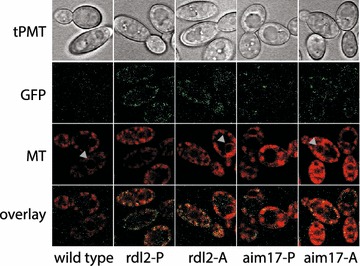
Mitochondrial marker co-localization with Mito-Tracker deep red. The cells of the two prototrophic mitochondrial strains both co-occur with the mitochondrial stain. The GFP signal is limited to mitochondrial membranes, while Mito-Tracker shows nonspecific signal around nuclei and other cytosolic structures, indicated by gray arrows. *tPMT* transmission PMT, *GFP* emission at 495–540 nm, *MT* Mito-Tracker deep red (Invitrogen), *overlay* GFP overlaid on Mito-Tracker

For all markers tested, localization in Y. lipolytica is consistent with S. cerevisiae orthologs and previous work [77, 78]. The function of the endoplasmic reticulum marker Emc2p is unrelated to lipid biogenesis, to avoid bias to a process of primary interest in *Y. lipolytica*. Emc2p is a member of a transmembrane complex; when members are deleted or perturbed, *S. cerevisiae* cells show signs of the unfolded protein response [79]. Vrg4p is a golgi GDP-mannose transporter needed for protein glycosylation [80]. We tagged two proteins predicted to localize to the nucleus. The linker histone H1 directly interacts with DNA throughout the interior of the nucleus while the ribosomal export protein Arx1p shuttles the pre-60S subunit for export, and interacts with a number of nucleoporin subunits [81, 82]. The primary localization of Arx1p-sfGFP is also nuclear, at times sub-localized, presumably at the nucleolus (Figs. 7, 8). We tagged two mitochondrial proteins. Rdl2p is a rhodanese-like protein which has thiosulfate sulfurtransferase activity [83]. Rdl2p function in *S. cerevisiae* is linked to H_2_O_2_ detoxification, which contributes to life span in longer-lived wine fermentation yeast strains, where this protein is highly expressed in the late stages of fermentation [84]. Aim17p is implicated in mitochondrial biogenesis in *S. cerevisiae* [85, 86]. Additionally, we report specific and active localization through the course of experiments to characterize these proteins with existing tools and techniques. Specific localization of the mitochondrial markers Rdl2p and Aim17p was more discrete than the commercially available MitoTracker (Fig. 9), which contains a reactive thiol and is delivered in dimethylsulfoxide to perforate cell membranes [87]. Nitrogen limitation in a carbon-replete environment is a condition used to stimulate lipid accumulation in microalgal, algal, cyanobacterial, and fungal organisms, including *Y. lipolytica*, for biofuel development [88–92]. We evaluated the atlas proteins for expression and organelle-specific localization in YPD and lower nitrogen (Y-D, YPD lacking peptone) media in both our prototroph and auxotroph strains (Figs. 7, 8). Localization not only was consistent with the expected organelle compartments but also reflects responses to different nutrient types. We found several interesting patterns after transfer to nitrogen-deficient (Y-D) medium that may be associated with stationary phase such as histone H1, important for DNA packaging [93]. Erg6 is a protein in the ergosterol biosynthetic pathway, used as a lipid droplet marker, and a target of antifungals [94, 95]. It has also been identified by proteomics in *Y. lipolytica* lipid droplets [56], and here shows changing lipid droplet size and number. Cyp1p, a nonspecific carboxypeptidase involved in protein degradation [96], also exhibits altered vacuole size and number consistent with a role in degradation. In total, we have tagged proteins covering seven cellular compartments.

### Mitochondrial response to reactive oxygen species is growth phase dependent

We wanted to assess the response of Rdl2p to hydrogen peroxide stress. It was previously observed that *S. cerevisiae* sensitivity to hydrogen peroxide is enhanced in minimal medium compared with the presence of amino acids and nucleobases [97], a susceptibility also confirmed in *Y. lipolytica* [98]. Further, it has been noted that prior exposure of yeast to an oxidative stress increases tolerance to higher concentrations of ROS (reactive oxygen species) [97] suggesting a physiological response which may be based in protein expression. ROS tolerance is also increased in stationary vs. exponential phase cells [99]. Susceptibility to and tolerance of oxidative stress is relevant to lipid biogenesis in several respects: lipids are a target for peroxidation by ROS, and the oxidative state of the cell may change the level of available NADPH, which is used directly in lipid biogenesis.

**Fig. 10:**
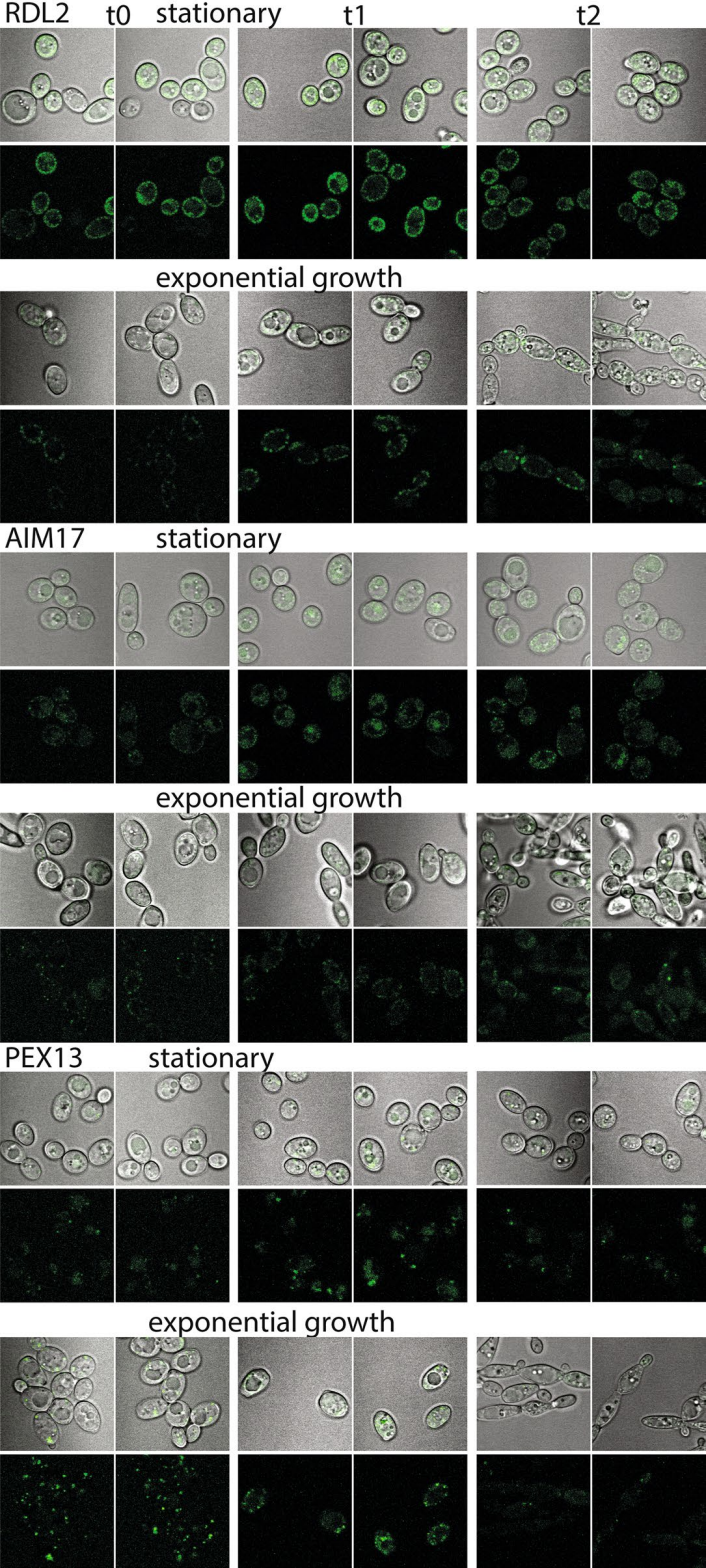
Mitochondrial and peroxisomal proteins under hydrogen peroxide stress. Organelle prototrophic atlas strains were grown for 16 h, after which an aliquot was removed to a fresh tube for the exponential samples. Live cells were imaged at time points t0 = prior to stressor, t1 = 1–2 h after transfer to 40 mM hydrogen peroxide YPD media, and t2 = 20 h after transfer. Images are internally normalized to the tagged protein in Fiji [136]

To determine the response of organelle tags involved in detoxification of ROS, we grew cells expressing Rdl2-sfGFPp, Aim17-sfGFPp, and Pex13-sfGFPp to exponential and stationary phases. They were then treated with hydrogen peroxide and imaged immediately and again after 24 h. We observed a difference in growth phase susceptibility, and that Rdl2-sfGFPp signal intensity changes after H_2_O_2_ treatment (Fig. 10). Stationary phase cells have emblematic mitochondrial signal, which is distributed as seen in other growth conditions and stains (Figs. 7, 8, 9) after 24 h of stress. Exponential phase cells are somewhat elongated in all samples, indicative of stress [100], and have punctate mitochondrial and peroxisomal protein signals. These results suggest that Rdl2-sfGFPp distribution, such as between exponential and stationary phase cells (Fig. 10), has the potential to be used as a relative marker of oxidative stress. Consistent with Rdl2 expression levels correlating with stress tolerance in *S. cerevisiae* [84], monitored expression of Rdl2 under controlled and experimental growth conditions will aid optimization of bioproduct production in this obligate aerobe.

### Pex13-sfGFPp signal is altered by carbon source and nitrogen limitation

The dynamics of *Y. lipolytica* peroxisomes have been followed by immunostaining [101, 102], by biochemical sedimentation gradients [13, 103], and mass spectrometry [104]. Expression of a limited number of peroxisomal protein components have been developed in other backgrounds [105, 106]. However, previously studied proteins are involved in beta-oxidation (Mfe1p), or are not a constant peroxisome resident (Pex3p) [106]. Malate synthase has been studied for alternative splicing, with one form localized by an N-terminal GFP to the peroxisome matrix by plasmid-based expression [107]. Peroxisome maturation has been studied in detail in *Y. lipolytica*, following a series of microbody structures which contain peroxisome associated proteins (Pex6p and Pex2p) and enzyme functions such as beta-oxidation and hydrogen peroxide detoxification [13]. Loss of peroxisome-localized beta-oxidation enzyme function resulting in increased lipid accumulation shows the importance of this organelle to biofuel development [108].

**Fig. 11:**
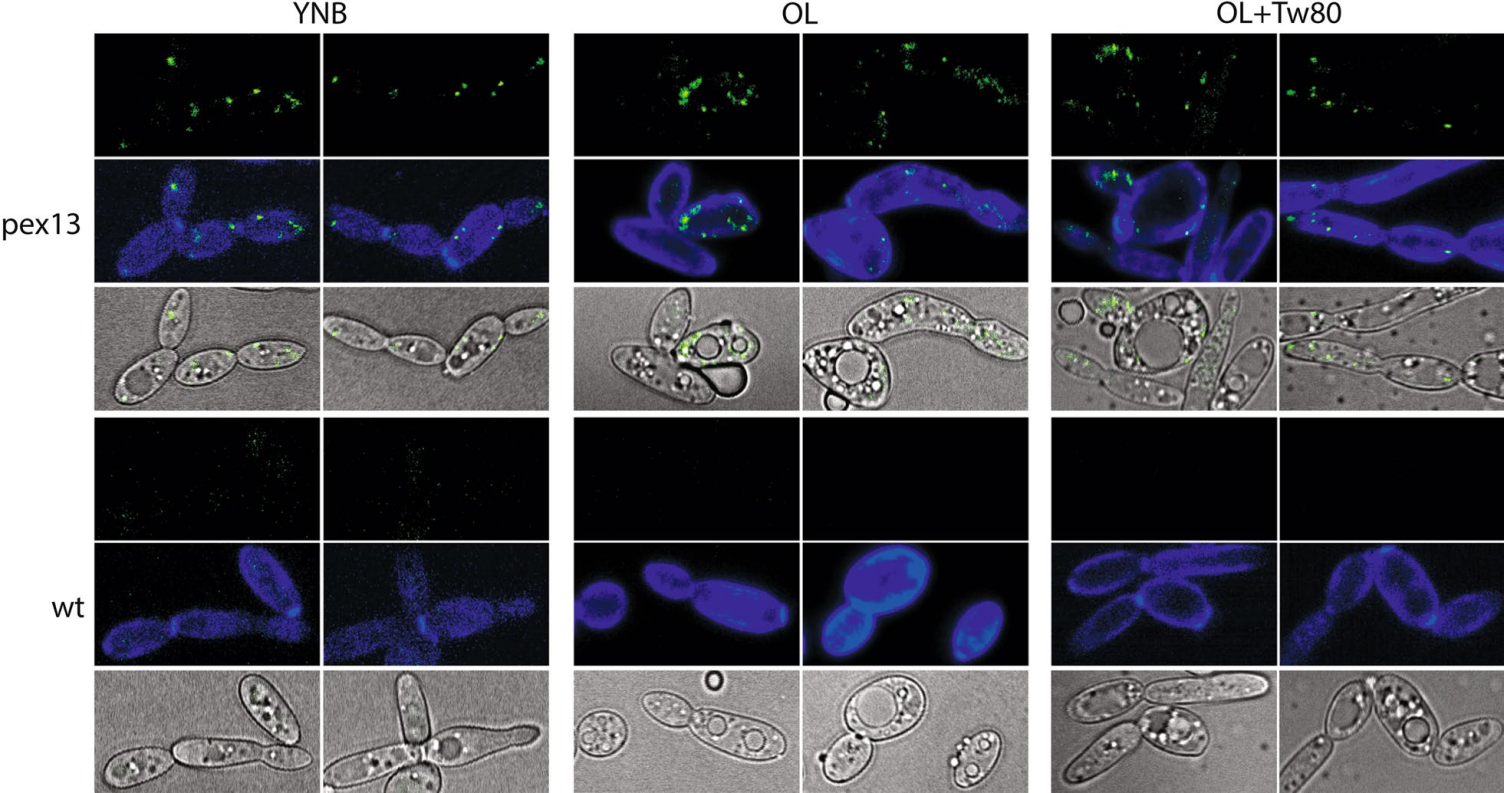
Peroxisome induction in carbon sources as visualized by pex13-gfp. Peroxisome development in untagged and pex13-GFP strains is followed showing specific localization. *Top images* GFP; *Middle images* GFP and calcofluor white staining; *Bottom images* transmission PMT overlaid with GFP

**Fig. 12:**
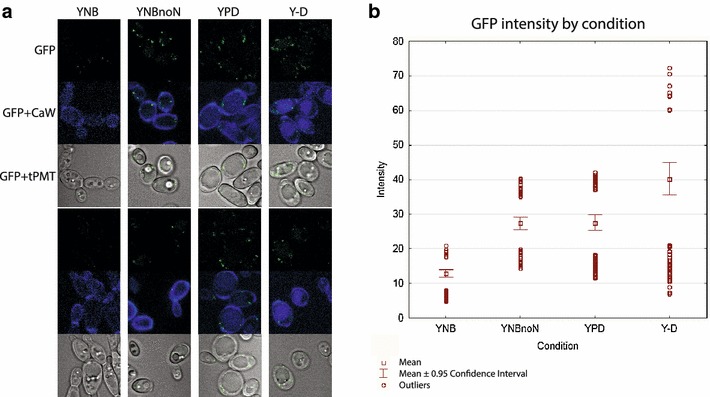
Pex13-GFP signal in media with nitrogen limitation. **a** Cells were inoculated from a single flask after growth into 10 mL each of 4 different media [YNB with 2% glucose and 0.5% ammonium sulfate (w/v), YNB with no ammonium sulfate, YPD, and yeast extract-dextrose with no peptone added (Y-D)]. The cultures were grown shaking at 200 rpm for 16 h followed by imaging on a Leica LSM710 scanning confocal microscope. *Upper* and *lower panels* are replicate images for each condition above. **b** Images were quantified for integrated intensity using CellProfiler [135] with calcofluor signal to find cell area, and statistical analysis done by Statistica. All groups are statistically different except YNBnoN and YPD

Pex13p in *S. cerevisiae* is a protein with a C-terminal SH3 domain used for docking peroxisomal targeting signal (PTS)-containing proteins, which are chaperones for peroxisome bound cargo [109, 110]. To demonstrate peroxisome labeling, Pex13-sfGFPp was grown with oleate as the sole carbon source (Fig. 11), which induces peroxisome formation. Pex13-sfGFPp signal is generally punctate but disperses somewhat after nitrogen limitation in the auxotrophic and prototrophic atlas strains (Figs. 7, 8). This suggests that Pex13p, an essential part of the protein import complex of Pex13/14/17 for movement into the peroxisomal matrix [111], may be redistributed or broken down in different media [105, 112]. To test this, we grew the Pex13-sfGFPp strain in rich medium (YPD) and then transferred it to rich and minimal (YNB) medium with and without (YNBnoN or Y-D) a nitrogen source for 16 h before microscopy. In both media types with reduced nitrogen, Pex13-sfGFPp signal is increased. Peroxisome signal is lost in minimal medium (YNB), and is greatest in nitrogen-deficient rich medium (Y-D) (Fig. 12). The signal quantification is in contrast to *S. cerevisiae*, which experiences induction of pexophagy by transfer from oleate media into glucose with nitrogen [113]. However, it is difficult to interpret total signal, as this could indicate redistribution or breakdown of the organelles. These data are evidence of different peroxisome behaviors in oleaginous organisms as compared to conventional yeast models. This strain set will allow minimization of peroxisome production and activity specific to *Y. lipolytica* without major biochemical pathway loss in the mutation of mfe1p.

## Conclusions

The cell biology of lipid accumulation is of industrial interest. Here, we have developed a suite of isogenic strains and codon optimized plasmids to enable cell biology studies in *Y. lipolytica*. We have sequenced, assembled, and annotated the initial non-homologous end-joining mutant strain used for construction of all the strains described herein allowing for accurate design of genetic constructs. Although some of the resources we have developed have been previously described in other genetic backgrounds in *Y. lipolytica* [31], including auxotrophs [7], utilization of *hph* [114] and *gfp* [115], and replicating and integrating plasmids, they have guided the design of these strains and plasmids. Having all these tools, along with the fluorescent organelle atlas in an isogenic non-homologous end-joining mutant will remove data differences caused by genetic background, and expedite functional genetics in *Y. lipolytica*. Organelle-specific GFP tagging can identify specific membrane structures under conditions relevant to industrial use, and parallel analysis in an isogenic background with live GFP-labeled proteins expressed from endogenous loci to monitor the size, shape, and quantity of a particular organelle. The atlas strains presented allow rapid and efficient targeted disruption, tagging, overexpression, multi-protein labeling with additional fluorescent tags, and an indicator of cellular stress as suggested by Rdl2-sfGFPp expression in response to reactive oxygen species (Fig. 10). Biofuel organism engineering efforts require consideration not only of total lipid accumulation, but also of cell health and growth rate. Continual drain on lipid resources may make senescence an issue, as the balance of triglyceride synthesis has been shown to confer an energy dependence increase in longevity to *S. cerevisiae* [116].

We present these tools as a compliment to the holistic approach on the global level of –omic analyses. The rapid increase in –omic studies in *Y. lipolytica*, which inquire multiple levels of regulation, has generated many hypotheses [15, 28, 117–119] that require more efficient tools for functional genomics. For example, during lipid accumulation experiments, membrane bound cellular substructures can be distinguished by confocal microscopy. While lipid droplets are frequently notable due to a difference in bright field diffraction, quantifying and evaluating their growth in live cells are a GFP functionality of interest for exploring lipid and metabolic phenotypes. We found previously that protein degradation, peroxisome activity, and alcohol sugar secretion were characteristics of *Y. lipolytica* during nitrogen starvation [28]. In order to study compartmentalized activities, we have included atlas strains demarking membrane-bound compartments relevant to these processes, including the vacuole (nitrogen storage and material degradation), mitochondria (producer of ATP and NADH and NADPH for lipid biosynthesis), golgi (protein modification, sorting, and secretion), peroxisome (oxidative detoxification, beta-oxidation), nucleus (transcriptional activity), and the endoplasmic reticulum (translation and lipid droplet biogenesis) which will enable new routes for the investigation of energy relevant organelles by the Yarrowia research community.

## Methods

### Chemicals

All chemicals and reagents were purchased from Thermo Fisher Scientific (Waltham, MA) unless otherwise noted. The water used was of Milli-Q grade purified by a Millipore (Bedford, MA) Milli-Q UV Purification System. PCR amplification was performed using Q5 high fidelity DNA polymerase (New England Biolabs; Ipswich, MA). Digestions used FastDigest enzymes (Thermo Fisher Scientific; Waltham, MA) and ligations used T4 DNA ligase (Life Tech.; Carlsbad, CA).

### Yeast strains and cultivation

Strains were maintained on YPD medium (1% yeast extract, 1% peptone, 2% glucose) or YNB medium (1.71 g/L Yeast Nitrogen base lacking amino acids, 5 g/L ammonium sulfate, 5 g/L glucose) at 28 °C unless otherwise noted. The widely used *Y. lipolytica* strain Po1g [7] is from Yeastern Biotech (Taipei, Taiwan). Wild-type *matA* (W29; ATCC20460™) and *matB* (CBS6124-2; ATCC18944™) *Y. lipolytica* strains were from American Type Tissue Culture (Manassas, VA).

### Generation of *ku70*::*hph* construct

To create the *ku70* gene deletion construct, 1.9 kb upstream and downstream of the *ku70* gene and 0.55 kb of the *Y. lipolytica tef1* promoter and the bacterial hygromycin phosphotransferase gene (*hph*) were amplified by PCR with oligo pairs (Additional file 3, Oligos) 1313/1314 (*ku70* upstream), 1315/1321 (*hph*), 1322/1318 (*tef1*), and 1319/1320 (*ku70* downstream), respectively. During PCR amplification, restriction sites for the endonucleases *Xho*I and *Hind*III were introduced at the 5′-end of *ku70* upstream and 3′-end of *ku70* downstream for further cloning. All four fragments were fused together by yeast gap repair with pRS426 vector as previously described [120] and then cloned into the T-DNA binary vector pZD663 (see Additional file 1: Figure S2). The fragment was confirmed by DNA sequencing. The pZD663 binary vector, a derivative of the pBI121 binary Ti plasmid [121], was constructed by replacing the whole DNA fragment between left and right borders of the pBI121 T-DNA region (GenBank: AF485783.1) with a synthetic DNA fragment containing ten unique multiple cloning sites.

### Agrobacterium-mediated transformation

The transgene expression T-DNA binary vector pZD663-ku70 downstream-tef1-hph-ku70 upstream was mobilized into the *A. tumefaciens* EHA105 strain by a freeze–thaw technique [122]. *A. tumefaciens* was grown in YEP (10 g/L yeast extract, 10 g/L peptone and 5 g/L NaCl) overnight. Agrobacterium cells were aliquoted into 5-mL induction medium [123] with and without 0.2 mM AS (acetosyringone) to a final density of 0.2 at A600 for an additional 5–6 h growth to final OD_600_ of 0.4–0.5 (~2 × 109 cells/mL determined by plate growth count). Three different amounts (5 × 10^6^, 1 × 10^7^, and 5 × 10^7^ cells) of overnight *Y. lipolytica* cells grown in YPD medium were aliquoted into microcentrifuge tubes and washed twice with IM buffer. Five different ratios of *Y. lipolytica* and Agrobacterium cells (i.e., 5 × 10^6^, 1 × 10^7^, and 5 × 10^7^ *Y. lipolytica*:100 µL (~2 × 10^8^) Agrobacterium cells with 0.2 mM AS; 1 × 10^7^ *Y. lipolytica*:300 µL (~6 × 10^8^) of Agrobacterium cells with 0.2 mM AS; and 1 × 10^7^ *Y. lipolytica*:100 µL (~2 × 10^8^) of Agrobacterium cells without AS) were mixed well in a final volume of 200 µL and spread onto the 25 × 30 mm sterile 0.45 µm Hybond-N + nylon membrane (GE Healthcare Bio-Sciences, Pittsburgh, Pennsylvania) laid on the IM agar plate with or without AS. After 2 days of incubation at room temperature (~23 °C), the transformed *Y. lipolytica* cells were washed from the nylon membrane with 5 mL sterile distilled water and 1/10 of the volume was spread onto the YPD agar plate with the 300 mg/L hygromycin B and 250 mg/L cefotaxime. The transformed *Y. lipolytica* cells were visible on the plate after incubation at 28 °C for 2 days. Individual colonies were picked and streaked onto a new YPD agar plate containing 300 mg/L hygromycin B and 250 mg/L cefotaxime (see Additional file 1: Figure S1). Single colonies were grown in 2 mL of YPD liquid medium containing the same set of antibiotics at 28 °C and 200 rpm for 18–24 h, which was used for strain storage and genomic DNA isolation.

### Identification of *Y. lipolytica ku70*::*hph* clones

The genomic DNA of selected *Y. lipolytica* transgenic clones was isolated by CTAB method [124] with modifications. Overnight cultures were transferred into a 2-mL screw-cap micro-tubes (Mikro-Schraubrohre) and centrifuged for 2 min at 17,000*g* and 20 °C. The supernatant was removed and 250 μL of CTAB and 250 µL of 0.5 mm zirconia/silica beads were added to each tube. The cells were homogenized in a mini-beadbeater (Biospec) for 2 min. The homogenized cell mixtures were incubated at 58 °C for 1 h. Genomic DNA was extracted with 170 µL of phenol:chloroform:isoamyl alcohol (25:24:1, pH 8.0) once and centrifuged for 8 min at 17,000*g*. After transfer, 500 μL of 95% ethanol was mixed with the supernatants, and incubated at room temperature for 10 min. The DNA was pelleted at 17,000*g* and 15 °C for 10 min, then re-suspended in 200 µL of 50× TE (50 mM TrisHCl/10 mM EDTA, pH8.0) containing 20 µg of RNase A per sample and incubated at 55 °C for 30~60 min. The DNA mixture was extracted twice with 200 µL of phenol:chloroform:isoamyl alcohol and centrifuged at 17,000*g* for 10 min. For precipitation, 20 µL of 3 M sodium acetate (pH 5.2) and 500 μL of 95% ethanol were added to each DNA sample, mixed gently and kept at room temperature for 10~30 min. The genomic DNA was pelleted by centrifugation at 10,000*g* and 15 °C for 10 min, then washed once with 1 mL of 70% ethanol. The dried DNA pellets were re-suspended with 50 µL of 10 mM Tris–HCl (pH 8.0). The genomic DNA was used for PCR screening with oligo pairs located outside the transgene deletion fragment of *ku70* (see Additional file 1: Figure S2B). The PCR product was digested with PvuII and separated by agarose gel electrophoresis to test for *ku70* gene replacement by *hph*. After screening about 120 individual transformants, we identified a clone in which *ku70* was replaced by *hph* (see Additional file 1: Figure S2C).

### Southern blotting analysis

For Southern blotting, one microgram of total genomic DNA from Po1g or *ku70*::*hph* strains was digested with the restriction endonuclease BglII, EcoRV, and PvuII, respectively. The genomic DNA fragments were separated by electrophoresis in a 1% agarose gel, then transferred onto the Amersham Hybond-N^+^ membrane (GE Healthcare Life Sci, Pittsburgh, PA) by alkaline capillary transfer. The 2.0 kb genomic DNA fragment of *ku70* gene upstream region used for the *ku70* deletion construct was used to prepare a biotin-labeled probe (see Additional file 1: Figure S2D). The genomic DNA on the Hybond-N^+^ membrane was hybridized with the biotin-labeled probe overnight at 60 °C in a Problot Hybridization Oven (Labnet International, Edison, NJ, USA) and visualized with a North2South chemiluminescent detection kit (Pierce Protein Research Products, Rockford, IL) in a Koda Imaging Station 2000R (Eastman Kodak Company, Rochester, NY, USA).

### Generation of isogenetic *ku70*::*hph* auxotrophs

*Ura3* mutants derived from FKP355 were selected on YNB with 5 g/L uracil and 5 g/L 5-FOA, and supplemented with 0.1 g/L leucine to make FKP393. *Leu2* was complemented in FKP355 resulting in FKP391 and also in FKP393, yielding FEB130 by transformation with a *leu2* PCR product amplified with primers OKP443 and OKP444.

### Genome sequencing, assembly, and annotation

Genomic DNA was isolated from FKP355 using the yeast genomic DNA purification kit (AMRESCO, Solon, OH) followed by 150 bp paired-end sequencing on an Illumina MiSeq instrument. The reads were assembled using Velvet v1.2.10 [125] with a k-mer length of 81 chosen to optimize for the highest *N*_50_. Raw RNA-seq data from previously described strains of similar genetic background [15, 51] were mapped to the assembled genome using TopHat v2.0.13 [126] to predict transcripts from which coding regions were predicted. Predicted proteins were functionally annotated using Blast2GO [127]. Full length transcripts were aligned to contigs from strain CLIB122 [44] using Blat v32×1 [128] to determine their position and percent identity. Reads were aligned to the reference genome using Bowtie v0.12.9 [129] and SNPs identified with custom Perl scripts.

### Plasmid construction

A codon optimized superfolder GFP (*sfGFP*) gene [37] followed by a codon optimized *hph* gene from *Escherichia coli* [130] driven by the high expression *Yarrowia lipolytica tef* promoter along with the centromere and origin of replication sequences from ARS68 [46] were synthesized and cloned into pMK-RQ to make pYL1. The high expression *exp1* promoter was amplified from *Y. lipolytica* strain Po1g with primer pairs OKP189/190 and ligated into pYL1 following digestion with KpnI and EcoRI to make pYL2 (Genbank: KU378203). The *Y. lipolytica leu2* gene was amplified with primers OKP193/194 and ligated into pYL2 following digestion with BamHI to make plasmid pYL4. Additional *leu2* 5′ flanking sequence was amplified with primer pair OKP451/452 and ligated into pYL4 following digestion with HindIII to make pYL15 (Genbank: KU378202). Homologs of enzymes predicted to be directly involved in the biosynthesis of triglycerides from glucose were predicted using BlastP. The coding region for each enzyme was PCR amplified from Y. lipolytica strain FKP355 genomic DNA using Q5 DNA polymerase (New England Biolabs, Ipswich, MA) and oligos (Life Technologies, Carlsbad, CA) designed with 5′ (5′-ATATCTACAGCGGTACCCCC-3′) and 3′ (5′-CCGCCTCCGCCGATATCCCCC-3′) overhangs homologous to plasmid pYL15 (listed in Additional file 3). Plasmid pYL15 digested with SmaI (Fermentas, Waltham, MA) and the PCR products were purified using a GeneJET purification kit (Thermo Fisher Scientific, Waltham, MA) and assembled using the NEBuilder HiFi assembly kit (New England Biolabs, Ipswich, MA) to produce the replicating plasmids listed in Additional file 4. FKP355 was transformed with pYL15 derived plasmids using the lithium acetate method [131] followed by selection on YNB agar. Transformants were verified by PCR and microscopy and maintained at −80 °C in 15% glycerol.

### Generation of organelle GFP library

Selection of candidate organelle-targeted proteins was done using quantitative and image data in *S. cerevisiae* GFP libraries [77, 78, 132]. For transformation, we utilized a PEG-lithium acetate buffer method developed for *S. cerevisiae* with modifications [133]. *Y. lipolytica* cells were grown for 1-3 days before transformation. DNA fragments of approximately 1 kb upstream (5′ flank) and downstream (3′ flank) of the stop codon of the gene for C-terminal GFP tagging were amplified (oligos used in Additional file 3). The *leu2* gene was amplified from pYL4 using OEB 170/171, and the codon optimized *sfGFP* from pYL2 using OKP31/OEB169 which append a synthetic linker for attachment to the selective gene. Targeted proteins selected by orthologous Blast (Table 4). Overlap PCRs were used to assemble the 5′ flank and *sfGFP* plus a partial region in the selection gene, and separately, another portion of the selection gene and the 3′ flank (OEB3/4 or OKP 211/212). These extension PCRs were run using a 15-cycle program to allow the fragments to prime against one another (98 for 2:00, with cycles of 98 for 0:30, 60 for 0:20, 72 for 2:00, followed by 72 for 5:00). Primers were added (5′ flank forward or 3′ flank reverse primer with the appropriate split marker primer), and the PCRs continued for 30 cycles. Gel purified fragments were extracted using a GeneJET Micro kit (K0832). Agar plates containing YNB with appropriate Leucine or Uracil supplementation (5 g/L) were used for selection [131, 133]. For organelle response to nitrogen limitation, strains were grown to turbidity shaking in YPD for 16 h (t0) followed by washing in YNB salts, and transfer to Y-D media lacking a nitrogen source for 20 h (t1).

### Microscopy

For microscopy, cells were visualized using a Zeiss LSM710 confocal laser-scanning microscope (Carl Zeiss MicroImaging GmbH, Munchen, Germany) with a Plan-Apochromate 100×/1.4 Oil objective. For co-localization studies, the cell cultures were stained with MitoTracker deep red (Molecular Probes-Thermo-Fisher, M22426, Eugene, OR) or lipidTOX red (Molecular Probes) according to the manufacturer’s instructions. Cells were grown overnight in 4 mL of YPD in a shake tube, then 700 μL was pelleted gently and transferred to 2 mL fresh YPD for 1 h containing MitoTracker Deep Red added to a 1:5000 dilution. Calcofluor white was added to the cells at the time of imaging with the addition of 1 μL of 1:150 dilution of a 1 mg/mL stock solution to every 4 μL of cells. Images were processed using imageJ [134] and CellProfiler [135]. Images were normalized within the tagged strain relative to wild-type or within time point as appropriate.

### Hydrogen peroxide stress

Atlas strains FEB64, FEB92, FEB93 were grown 16 h in YPD with shaking. For exponential phase cells, 1 mL of culture was transferred to fresh media, while the remaining stationary cells were used in parallel at the 24 h mark for stress treatment. Briefly, cells were collected by centrifugation at 200 rpm for 1 min. Reserving some of the initial culture for imaging (t0), strains were transferred to YPD containing 40 mM hydrogen peroxide, then imaged at 1–2 h (t1) and 20 h (t2).

### Peroxisome media assay

Strain FEB64 was grown overnight, collected by centrifugation, washed 1X in PBS, and then equally inoculated into 10 mL of rich (YPD), minimal (YNB; 1.7 g/L yeast nitrogen base without amino acids and ammonium sulfate, 20 g/L glucose, 5 g/L ammonium sulfate), nitrogen-deficient rich (Y-D; 10 g/L yeast extract, 20 g/L glucose) and nitrogen-deficient minimal medium (YNBnoN; 1.7 g/L yeast nitrogen base without amino acids and ammonium sulfate, 20 g/L glucose). Cultures were grown shaking at 200 rpm for 16 h, and then imaged. Calcofluor staining was used for definition of cellular space in Cell Profiler.

## Additional files

**Additional file 1.** Supplemental figures.

**Additional file 2.** Protein annotations of FKP355.

**Additional file 3.** Oligos used for PCR.

**Additional file 4.** Plasmids built and used in this study.

## Abbreviations

ACC: acetyl-CoA carboxylase
ACL: ATP-citrate lyase
AS: acetosyringone
bp: base pair(s)
Mb: megabases
CDS: CDP-Diacylglycerol Synthase
GFP: green fluorescent protein
sfGFP: superfolder green fluorescent protein
FKP: FEB-fungal strain numbers by author initials
hph: hygromycin phosphotrasferase, gene confers resistance to hygromycin B
PTS: peroxisome targeting signal
ROS: reactive oxygen species
SNP: single nucleotide polymorphism
YPD: yeast extract, peptone, dextrose media, see “Methods” section
YNB: yeast nitrogen base media, see “Methods” section
