# Erratum to: A molecular genetic toolbox for *Yarrowia lipolytica*

**DOI:** 10.1186/s13068-017-0731-2

**Published:** 2017-02-22

**Authors:** Erin L. Bredeweg, Kyle R. Pomraning, Ziyu Dai, Jens Nielsen, Eduard J. Kerkhoven, Scott E. Baker

**Affiliations:** 1 Earth and Biological Sciences Directorate, Environmental Molecular Sciences Laboratory, Richland, WA 99354 USA; 2 Chemical & Biological Process Development Group, Energy and Environment Directorate, Pacific Northwest National Laboratories, Richland, WA 99354 USA; 3 Systems and Synthetic Biology, Department of Biology and Biological Engineering, Chalmers University of Technology, Göteborg, Sweden; 4 Novo Nordisk Foundation Center for Biosustainability, Technical University of Denmark, Hørsholm, Denmark; 5 Department of Energy, Battelle EMSL, 3335 Innovation Blvd, Richland, WA 99354 USA

## Erratum to: Biotechnol Biofuels (2017) 10:2 DOI 10.1186/s13068-016-0687-7

Shortly after this article was published [1] the authors noticed a few errors which are outlined below:

Background paragraph 1, sentence 4, The wild type American CBS number should be: “CBS6124-2.”

Background paragraph 1, sentence 5 should be: “The Po1 series, derived directly of successive transformations of W29 [7], has been used for a number of studies.”

Background paragraph 1, sentence 6 should be: “CLIB122 or E150, the reference genome strain, is derived of a W29 to YB423-12 cross and brother–sister matings, *xpr2* deletion, and introduced auxotrophies [8].”

Background paragraph 1, sentence 7 should be: “Genome sequencing efforts have covered some original isolates and their derivatives, including W29 [9], and Po1f, a product of successive transformations [10].”

Page 13, Results paragraph 10, sentence 4 should be: “Malate dehydrogenase has been studied for alternative splicing, with one form localized by an N-terminal GFP to the peroxisome matrix by expression from an integrated cassette [107].”
