# Supplementary material for: A molecular genetic toolbox for Yarrowia lipolytica
Source: Biotechnol Biofuels. 2017 Jan 3;10:2. doi: 10.1186/s13068-016-0687-7 (PMC5210315; doi:10.1186/s13068-016-0687-7)
Supplement: Supplementary file 1 — Additional file 1. Supplemental figures. [file 13068_2016_687_MOESM1_ESM.doc]

**Figure S1: Hygromycin resistance of different wild type strains.**

W29 is the upper left, Po1g is the upper right, ATCC18944^TM^ (bottom center) shows the ability to adapt to concentrations of hygromycin B up to 250 ug/mL. Concentrations of Hygromycin B (mg/mL) in YPD plates are left to right: 0, 62.5, 125, 250, 500).

**Figure S2: Vector construction and Southern of *ku70* knockout.**

A. Schematic of the plasmid including the left and right boundaries constructed to have multiclonal sites. B. Diagram of PvuII sites in the wild type and *ku70* mutant constructs C. PCR of *ku70* locus from several transformants showing the *ku70* mutant. D. location of southern probe E. Southern blot confirming single, correct integration of *ku70* mutant construct.
